# A Central Nervous System Focused Treatment Program for People with Frozen Shoulder: A Feasibility Study

**DOI:** 10.3390/ijerph19052628

**Published:** 2022-02-24

**Authors:** Silvia Mena-del Horno, Lirios Dueñas, Enrique Lluch, Adriaan Louw, Alejandro Luque-Suarez, Michel GCAM Mertens, Laura Fuentes-Aparicio, Mercè Balasch-Bernat

**Affiliations:** 1 Department of Physiotherapy, University of Valencia, 46010 Valencia, Spain; silvia.mena@uv.es; 2 Facultad de Ciencias de la Salud, Universidad Internacional de Valencia, 46002 Valencia, Spain; 3 Physiotherapy in Motion, Multi-Specialty Research Group (PTinMOTION), Department of Physiotherapy, University of Valencia, 46010 Valencia, Spain; enrique.lluch@uv.es (E.L.); laura.fuentes@uv.es (L.F.-A.); merce.balasch@uv.es (M.B.-B.); 4 Evidence in Motion, San Antonio, TX 16414, USA; adriaan@eimpt.com; 5 Physical Therapy Department, St. Ambrose University, Davenport, IA 52803, USA; 6 Facultad Ciencias de la Salud, Universidad de Málaga, 29071 Málaga, Spain; aluques@uma.es; 7 Instituto de la Investigación Biomédica de Málaga (IBIMA), 29010 Málaga, Spain; 8 MOVANT Research Group, Department of Rehabilitation Sciences and Physiotherapy, Faculty of Medicine and Health Sciences, University of Antwerp, 2610 Wilrijk, Belgium; michel.mertens@uantwerpen.be

**Keywords:** adhesive capsulitis, feasibility study, frozen shoulder, motor imagery, patient compliance, tactile discrimination training

## Abstract

Background: Frozen shoulder (FS) is a highly disabling pathology of poorly understood etiology, which is characterized by the presence of intense pain and progressive loss of range of motion (ROM). The aim of this study is to evaluate the feasibility and clinical impact of a CNS-focused treatment program for people with FS. Methods: 10 subjects with primary FS received a 10-week CNS-focused intervention including sensory discrimination training and graded motor imagery techniques delivered as clinic sessions (60 min) and home therapy (30 min five times per week). Measurements were taken at baseline, after a 2-week “washout” period, after treatment, and at three months follow-up. The Shoulder Pain and Disability Index (SPADI) was the primary outcome. Secondary measures were feasibility-related outcomes, self-reported shoulder pain, active and passive range of motion, two-point discrimination threshold (TPDT), left/right judgement task (LRJT), fear-avoidance (Tampa Scale for Kinesiophobia), pain catastrophization (Pain Catastrophizing Scale), and pain sensitization (Central Sensitization Inventory). A Student’s *t*-test was used to assess the “washout” period. A repeated measure analysis of variance (ANOVA) was used to evaluate within-subjects’ differences for all outcome measures in the different assessment periods and a pairwise analysis was used to compare between the different assessment points. Statistical significance was set at *p* < 0.05. Results: 70% of participants completed the treatment. No significant changes were found after “washout” period except for TPDT (*p* = 0.02) and SPADI (*p* = 0.025). Improvements in self-reported shoulder pain (*p* = 0.028) and active shoulder flexion (*p* = 0.016) were shown after treatment (*p* = 0.028) and follow-up (*p* = 0.001) and in SPADI at follow-up (*p* = 0.008). No significant changes were observed in TPDT, LRJT, fear-avoidance, pain catastrophization, and pain sensitization. Conclusions: a CNS-focused treatment program might be a suitable approach to improve pain and disability in FS, but further research is needed to draw firm conclusions.

## 1. Introduction

Frozen shoulder (FS) is a highly disabling pathology of poorly understood etiology [1], which is characterized by the presence of intense pain and progressive loss of range of motion (ROM) [2]. FS is present in 2–5% of the general population, especially in women aged between 40 and 65 years and its exact etiology is currently unknown [3]. The pathophysiology of FS is a complex and multifactorial process encompassing several mechanisms such as an upregulation of grown factors and inflammatory cytokines, which stimulate fibroblast proliferation and differentiation into myofibroblasts. This in turn leads to an imbalance of extracellular matrix turnover and a resultant stiff and thickened glenohumeral capsule with an abundance of type III collagen [4]. Accumulation of advanced glycation end products (AGEs) has also been shown in people with FS [5]. In addition, a state of low grade inflammation, which is associated with diabetes, cardiovascular disease, and thyroid disorders, seems also to predispose to the development of FS [6]. Many treatments have been proposed for FS including conservative (i.e., manual therapy) [7] and non-conservative approaches (i.e., arthroscopic capsular release) [8]. The most common and recommended physical therapy interventions used for treating these patients are mobilization techniques and exercises, while the utility of other suggested interventions such as aerobic exercise, lifestyle changes, or pain neuroscience education is still hypothetical [9]. To date, none of these interventions has demonstrated to have an influence on the natural history of this condition, therefore innovative research seems necessary [10]. Some authors have suggested an involvement of central pain mechanisms secondary to continuous nociception characteristic of the early stages of FS [10]. In line with this, two systematic reviews showed preliminary evidence that central pain mechanisms may contribute to shoulder pain of different etiologies [11,12], but recent studies questioned those findings [13,14]. Importantly, these reviews did not include people with FS, so the role of the central nervous system (CNS) in this clinical condition remains speculative. 

Different approaches targeting the CNS (e.g., graded motor imagery (GMI) and tactile discrimination training) have been applied in a variety of chronic musculoskeletal pain disorders with promising results [15,16]. Specific to shoulder pain, only a few studies have investigated the clinical effectiveness of CNS-focused interventions. Louw et al. [17] presented a case-series where a CNS-focused treatment program based on a brief mirror therapy intervention was applied in subjects with shoulder pain and limited active ROM. This approach showed statistically significant improvements in pain, pain catastrophization, fear-avoidance, and shoulder flexion active ROM [17]. However, only 8.7% of the sample presented a diagnosis of FS. Similarly, Sawyer et al. [18] applied a combination of pain neuroscience education, tactile discrimination training, and GMI in an individual with FS. The patient reported significant improvements in pain, fear of movement, and active ROM. Further high-quality research about the effectiveness of CNS-focused treatments in people with FS is thus needed. 

The aim of this study is to evaluate the feasibility and clinical impact when implementing a CNS-focused treatment program for people with FS. The results of this study will inform of the appropriateness to conduct a randomized controlled trial on this topic.

## 2. Materials and Methods

### 2.1. Sample Recruitment

A convenience sample of 10 subjects diagnosed with FS was recruited. Since there is no gold standard to diagnose FS, diagnosis was established by a physician based on clinical examination, exclusion of other pathologies, and imaging [19]. Patients included had to present with primary or idiopathic FS, a limitation in passive external rotation >50% compared to the unaffected shoulder or less than 30° of passive external rotation, and a ROM loss >25% in at least two movement planes [20]. Additionally, pain and movement restriction had to be present for at least one month having either reached a plateau or worsened [20] and radiographs had to be normal (with the exception of osteopenia of the humeral head and calcific tendinosis) [21]. 

Patients that presented with locked dislocations, arthritis, fractures, or avascular necrosis were excluded. Furthermore, those subjects not understanding Spanish language, having previous upper quadrant region surgery during the last year, any skin or medical condition preventing them from receiving tactile stimuli on the shoulder, any neurological or motor disorder, visually impaired, or having a diagnosed psychopathology were excluded from the study. All participants were instructed to continue taking any current medications, but not to start new medications or initiate new treatments during the treatment period. 

### 2.2. Procedures

**Figure 1:**
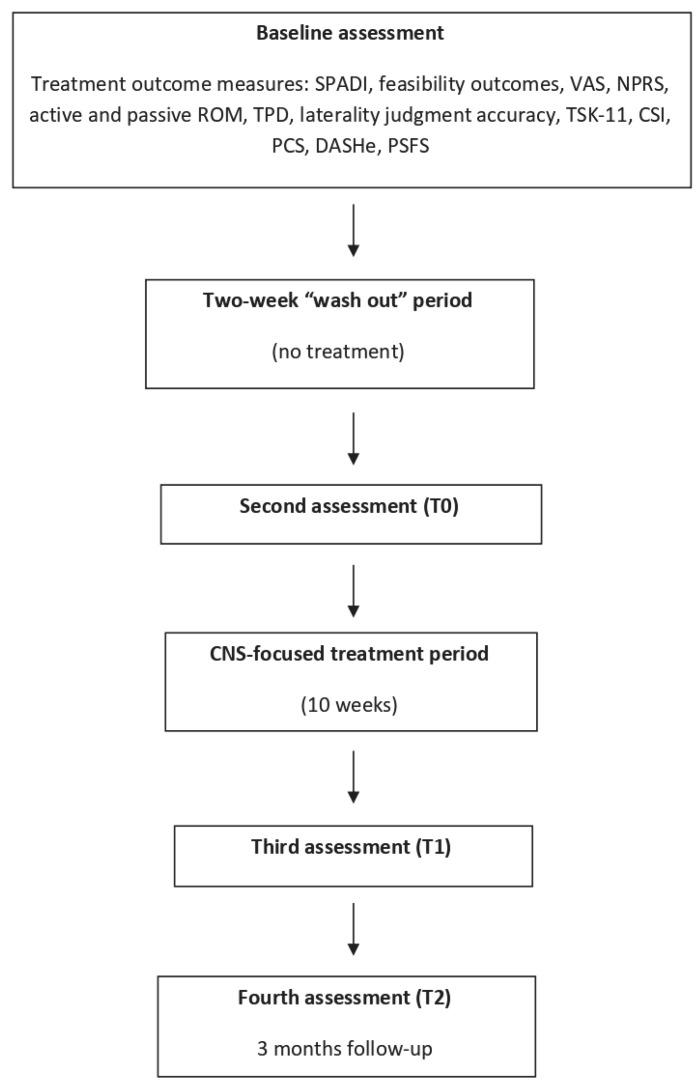
Assessment and treatment flowchart diagram.

This feasibility study involved a 10-week CNS-focused intervention and periodic assessment of the participants. All outcome measurements were performed at baseline and after a two-week period of “washout” with no intervention (T0) [22]. After this initial assessment, participants began the treatment and were again measured at the end of treatment (3 months after baseline (T1) and at three months follow-up (T2) (Figure 1)).

The CNS-focused intervention consisted of a 10-week treatment program (1 session per week) delivered as 60 min sessions. In addition, participants performed a 30-min home training program five times per week during those 10 weeks. The CNS-focused intervention included discussion of the participant’s shoulder pain experience from a pain neuroscience perspective provided in the first session plus graded sensory discrimination training and GMI [23]. The physiotherapist performing treatment (S.M.) had a post-graduate degree in manual therapy and was trained in how to perform the treatment by another researcher (E.LL.) with 10 years working experience in the use of these interventions.

### 2.3. Primary Outcome Measure

The primary outcome was self-reported shoulder pain and disability measured with the Shoulder Pain and Disability Index (SPADI) [24]. The SPADI is a 13-item shoulder function index assessing pain and disability related to shoulder dysfunction [25]. Each item is scored by a numeric scale (0–10) and the total score ranges from 0 to 100 points. A higher score indicates greater disability. The Spanish version of the SPADI has shown high internal consistency and excellent test-retest reliability [26]. The Minimal Clinically Important Difference (MCID) for the SPADI ranges from 8 to 13 points [27].

### 2.4. Secondary Outcome Measures

Different feasibility outcomes were considered as secondary: timely recruitment, number of participants completing treatment, treatment compliance and barriers (with clinic and home training sessions), and number of patients measured at follow-up. To assess treatment adherence, patients were provided with a diary to record their compliance with therapy [28]. After treatment completion, patients provided the diary to the physiotherapist performing the intervention to monitor adherence to the home training program for later analysis. In addition, patients were asked whether any difficulties with treatment compliance had appeared from one session to another. Additionally, other secondary outcome measures were collected: self-perceived shoulder pain, active and passive ROM, tactile acuity and laterality judgement performance, Tampa Scale for Kinesiophobia (TSK-11), Central Sensitization Inventory (CSI), and Pain Catastrophizing Scale (PCS).

#### 2.4.1. Self-Perceived Shoulder Pain

Participants’ self-perceived shoulder pain was evaluated with the Numeric Pain Rating Scale (NPRS) anchored between 0 (“no pain”) and 10 (“pain as bad as you can imagine”). Patients reported their most intense pain over the last week, least intense pain over the last week, average pain intensity over the last week, and pain at that moment. The scores were averaged to calculate a final pain intensity score [29]. NPRS is a valid and reliable measure in patients with shoulder pain [30]. The minimal detectable change (MDC) of the NRPS for patients with shoulder pain is 2.5 points and the MCID is 1.1 points [30].

#### 2.4.2. Shoulder Range of Motion

Shoulder flexion and active and passive external rotation at 0° of abduction of the affected shoulder were measured with a goniometer with the patient seated. To allow consistency of pre- and post-therapy measurements, skin marks were placed for goniometric measurements. A good reliability and validity of goniometric shoulder ROM measurements has been previously reported [31]. The MDC for shoulder flexion, abduction, and external rotation ranges from 11° to 16° [32].

#### 2.4.3. Tactile Acuity

Tactile acuity was assessed with the two-point discrimination threshold (TPDT). A mechanical sliding calliper with a 1-mm precision (Duratech TA-2081) was used to calculate the TPDT. Participants were placed in a sitting position and a point 5 cm distal to the lateral border of the acromion was marked on the painful shoulder. In order to standardize the testing region, this point was always kept between the two calliper points and measurements were performed in the longitudinal direction of the arm [33]. An ascending and a descending run of measurements were completed. The calliper distance was first gradually increased from 0 mm in 5 mm steps until the participant perceived two points instead of one. The descending run began with the calliper points separated 30 mm more than the TPDT value obtained from the ascending run, followed by decrements of 5 mm. A mean TPDT value was obtained from the two threshold scores and used for analysis. 

#### 2.4.4. Laterality Judgement

Laterality judgement was assessed with a left/right judgement task (LRJT) using the NOI™ online program. A total of 30 shoulder pictures (context mode) were presented to participants on a laptop in a random order and they were instructed to decide as quickly as possible, but without guessing, whether the picture showed the right or left shoulder thus making a response. Accuracy and mean response time were recorded. The LRJT was performed twice. The first block of images was used for task familiarization and data from the second block was used for analysis [34]. The normative mean (SD) response time and mean (SD) accuracy of this LRJT is 1738 (741) ms and 93.5 (9.2)%, respectively [35].

#### 2.4.5. Questionnaires

Fear-avoidance was assessed with the Spanish version of the TSK-11 [36]. The TSK-11 is an 11-item questionnaire used to assess fear of movement or (re)injury during movement [37]. The total score ranges from 11 to 44, with higher scores indicating more fear-avoidance behavior. The TSK-11 has shown acceptable internal consistency and validity in both subjects with acute and chronic musculoskeletal pain [36]. The MDC for the TSK-11 is 5.6 [38]. The Spanish version of the CSI was used to assess different symptom dimensions related to central sensitization [39]. The CSI has high test-retest reliability and internal consistency [39]. Moreover, pain catastrophization was assessed with the Pain Catastrophizing Scale (PCS). PCS consists of 13 items and the total score ranges from 0 to 52 [40]. A total PCS score of 30 represents a clinically relevant level of catastrophizing [40].

### 2.5. CNS-Focused Treatment Program 

Prior to starting treatment, participants were given an explanation of the study. Patients were shown a picture of the ‘brain map’ (homunculus) and taught how, when people are in pain, the map becomes “less sharp” since it is not being moved and it is believed that when the map is sharpened, it may help reduce their pain and even movements [17]. By using sensory discrimination training and GMI, the therapy aimed to sharpen the brain shoulder map and thus improve pain and movement. The CNS-focused treatment included graded sensory discrimination training and GMI training techniques. A full description of the treatment can be found elsewhere [41].

### 2.6. Statistical Analysis 

Statistical analysis was conducted using IBM SPSS Statistics 21. Normality of the data was assessed using the Shapiro–Wilk test. Study findings are expressed as the mean and standard deviation or 95% confidence interval, or as percentage frequencies. A Student’s *t*-test was used to assess differences between baseline and T0 (“washout” period). A repeated measure analysis of variance (ANOVA) was used to evaluate within-subjects’ differences for all outcome measures in the different assessment periods and a pairwise analysis was used to compare between the different assessment times. Statistical significance was set at *p* < 0.05.

## 3. Results

### 3.1. Participants’ Clinical and Demographic Data

The clinical and demographic characteristics of the participants at baseline are presented in Table 1. Only three patients (1, 8, and 9) presented moderate levels of pain (NPRS ≤ 5). Symptom duration ranged between two months and two years. Three patients (3, 8, and 10) demonstrated impaired tactile acuity (i.e., larger TPDT) at baseline in the affected shoulder compared to normative values reported for healthy individuals [i.e., 44.8 (13.1) mm] [33]. A total of 80% of the subjects presented lower accuracy in the LRJT at baseline compared to normative values [35]. This lower accuracy was observed bilaterally in 50% of the subjects and in the affected side in 30%. Only two patients (1 and 8) were slower in the LRJT in the affected shoulder compared to normative values [35]. Six patients were slower in the LRJT in the non-dominant shoulder.

### 3.2. Primary Outcomes

The SPADI scores improved after treatment in the different assessment times (*p* = 0.001). Significant changes in SPADI scores between baseline and follow-up (baseline-T2) (*p* = 0.008), but not between baseline and post-treatment (baseline-T1) or between post-treatment and follow-up (T1-T2) were observed (Table 2).

### 3.3. Secondary Outcomes 

Seven participants (70%) completed the treatment and all the measurements. The three patients (3, 5, and 8) not completing the treatment attended three, four, and six sessions, respectively. They dropped-out due to either difficulty for assisting to clinic sessions or lack of support from relatives to comply with home training. No adverse effects were found during or after the intervention. All patients completed the daily treatment diaries consistently. 

No significant changes were found after the “washout” period for all outcome measures except for TPDT (*p* = 0.02) and SPADI (*p* = 0.025). A significant decrease in shoulder pain was found after treatment (*p* = 0.028), between post-treatment and follow-up (*p* = 0.028), and between baseline and follow-up (*p* = 0.004) (Table 3). Significant improvements were found for active shoulder flexion (*p* < 0.001). 

Additionally, a significant improvement in active shoulder flexion after treatment (*p* = 0.016), between post-treatment and follow-up (*p* = 0.020), and between baseline and follow-up (*p* = 0.001) was found (Table 3).

There were no significant changes in tactile acuity or laterality judgement performance over time (Table 4). No significant changes were found in TSK-11, PCS, or CSI at any assessment time.

## 4. Discussion

The main goal of this study was to evaluate the feasibility of implementing a CNS-focused treatment program for people with FS. Furthermore, we aimed to assess the clinical impact of this program on pain and function. Overall, no significant changes were found after the “washout” period thus suggesting minimal changes in the participants’ clinical condition before treatment. Our findings revealed medium adherence of participants (70%) to the CNS-focused treatment and follow-up measurements. Regarding clinical impact, improvements in shoulder pain and active shoulder flexion were shown after treatment and at three months follow-up and in disability at three months follow-up. No significant changes were observed in tactile acuity, laterality judgement, pain catastrophization, fear-avoidance, or central sensitization after treatment or at follow-up. 

Average participants’ compliance with treatment was lower than expected. Participants’ compliance was recorded with a treatment diary which was consistently fulfilled by all participants, but it was not enough for them to comply with the totality of treatment as previously reported by Moseley et al. [28]. Nevertheless, all participants who attended the totality of treatment sessions at the clinic also met the home training dosage. In the current study, drop-outs were mainly due to a lack of support from relatives to assist participants with their home training tasks. Previous studies have also emphasized the difficulties with implementing CNS-focused techniques, in particular home training tasks, due to the lack of “helpers” availability or lack of time from participants [22,42]. These findings highlight the importance of having a cooperative context when using this kind of therapeutic approach at home. Long-term follow-up of participants was almost feasible as eight participants were followed-up. Only two participants were lost to follow-up, as they decided to discontinue the clinical sessions due to difficulties in the conciliation of their work schedules or lack of assistance with home training tasks. 

Regarding clinical outcomes, positive effects on pain and shoulder function were observed after treatment, which is in accordance with previous studies using a similar protocol [18]. Specifically, improvements were found in shoulder pain and active shoulder flexion both after treatment and follow-up measurements and in disability scores at follow-up. Regarding disability, the change in SPADI scores at follow-up exceeded both the MDC and MCID established for individuals with FS and non-specific shoulder pain, respectively [27,43]. Likewise, changes in pain intensity after treatment and at follow-up and in active shoulder flexion after treatment and at follow-up also surpassed the MCID established for pain intensity (1.1 points) and MDC for active shoulder flexion (11°) in people with shoulder pain, respectively [30,32]. No significant changes were found in LRJT and TPDT neither after treatment nor at follow-up. To our knowledge, responsiveness to treatment of these two variables in people with FS had not been previously investigated except in a single case report [18], where a 10 mm TPDT reduction and improvement of accuracy and response time in the LRJT task were observed after intervention. A case-series study [44] investigated the efficacy of a treatment combining GMI with mirror therapy in five patients with different shoulder painful conditions, including one patient with FS. After treatment, all patients showed significant improvements in pain intensity, active shoulder flexion, and motor imagery ability, but no significant changes on laterality judgement were found. 

No significant changes in fear-avoidance or pain catastrophization were found after treatment. This is not surprising given the nature of the CNS-focused treatment program, which mainly included sensory discrimination training and GMI. These two interventions were not expected to address fear or pain catastrophization. In this regard, pain neuroscience education has demonstrated clinically relevant effects in reducing psychosocial factors, in particular kinesiophobia and pain catastrophizing [45], but only a short discussion of pain from a pain neuroscience perspective was implemented in this study. This may explain the lack of change in psychosocial variables. Future studies could explore the role of pain neuroscience education in this population as recently recommended by some authors [9].

On the other hand, the duration of symptoms of our sample spanned over a wide range (2–24 months), meaning that participants may have entered the study at different stages of the disease. It is known that larger improvements in the natural history of FS are often found in the early stages of the disease (e.g., during the first year) [46]. The results of the current study cannot determine whether this CNS-focused approach would be more suitable to subjects with FS either in their early or late stage of the disease.

To our knowledge, a CNS-focused treatment had not been used before specifically for people with FS, except in a case report [18]. However, the aforementioned study did not include home training sessions. In contrast, the present study integrated both clinic and home training sessions, which was considered essential to properly investigate the feasibility of applying this kind of approach in clinical practice. 

## 5. Study Limitations

Our results need to be interpreted in light of some limitations. This feasibility study recruited a sample of only ten participants with FS. Despite the reported significant improvements in pain, disability, and ROM, clinical effects must be interpreted with caution as a greater sample of participants is needed to better estimate the utility of this treatment for people with FS. Another important limitation is the lack of a control group with no intervention, which has not allowed to reveal the natural history of FS, so future research should overcome this issue. 

Moreover, the heterogeneity of the recruited participants at baseline in terms of pain intensity and symptom duration limits the generalization of our results.

As participants completed the questionnaires alone and not in the presence of any researcher, this may have been one of the causes of the observed drop-outs.

Even though participants were allowed to continue with their current medication, the presence and absence of concomitant treatments, including specific medication intake, was not recorded. How these concomitant treatments may have influenced the results of this study is unknown.

Overall, this study identified key feasibility issues related to home training compliance that should lead one to reflect when using this approach, especially concerning the need of support from relatives. 

## 6. Conclusions

The results of this feasibility study suggest that a CNS-focused treatment program might be a suitable approach to improve pain and disability in people with FS, but further research with a greater sample of participants is needed to draw firm conclusions. Although a high percentage of the sample completed the whole treatment program, some fulfillment issues arose, such as the need for the patient to have a cooperative context when implementing this treatment at home.

## Figures and Tables

**Table 1:** Clinical and demographic characteristics of the participants at baseline.

Patient											
		1	2	3	4	5	6	7	8	9	10
Age (years)		51	51	49	49	46	63	59	58	48	47
Sex (male/female)		f	f	f	f	f	f	m	f	f	M
Weight (kg)		53	57	85	55	55	74	60	63	63	75
Length (cm)		169	164	175	166	155	164	170	162	168	189
Affected shoulder		left	right	right	right	right	right	right	left	left	left
Dominant Side		right	right	right	right	right	right	right	right	right	right
Symptoms duration (months)		2	15	6	6	16	12	3	3	24	10
SPADI (0–100)		91.54	26.15	20	59.23	20	74.62	40.77	75.38	62.31	54.62
NPRS (0–10)		5	2	1	3	3	0	1	5	5	2
PER ROM(degrees)		6	24	34	0	56	55	14	28	18	43
AF ROM (degrees)		60	110	102	66	156	150	86	78	118	140
TPD threshold (mm)		22.5	35	120	37.5	35	20	27.5	50	20	57.5
Left/right accuracy (%)	Left	87	100	100	100	100	73	93	93	100	93
	Right	87	93	80	10	80	67	87	73	100	93
Left/right speed (s)	Left	1.8	1.9	1.8	1.8	2.2	2	1.4	2.5	1.2	1.6
	Right	2	1.6	1.2	1.4	1.4	1.4	1.3	1.7	1.3	1.8
PCS (0–52)		11	4	0	2	35	13	23	18	19	18
CSI (0–100)		47	16	29	16	54	36	21	45	15	10
TSK-11 (11–44)		35	16	15	15	32	21	27	20	33	36

SPADI, Shoulder Pain and Disability Index; NPRS, Numeric Pain Rating Scale; PER, passive external rotation; AF, active flexion; TPDT, Two Point Discrimination Threshold; PCS, Pain Catastrophizing Scale; CSI, Central Sensitization Inventory; TSK-11, Tampa Scale for Kinesiophobia.

**Table 2:** Questionnaires results at baseline, two-week “washout” period (T0), post treatment (T1), and follow-up (T2).

		Mean ± SD	MD
SPADI (0–100)	Baseline	47.6 ± 25	
	T0	52.4 ± 24.9	4.8
	T1	31.6 ± 31.5	−16
	T2	19.4 ± 24.5 #	−28.2
TSK-11 (11–44)	Baseline	23.9 ± 8.3	
	T0	23.6 ± 8	−0.3
	T1	19.9 ± 8.5	−4
	T2	19.4 ± 8.9	−4.5
CSI (0–100)	Baseline	28.9 ± 15.7	
	T0	28.8 ± 14.7	−0.1
	T1	24.4 ± 13.04	−4.5
	T2	21.9 ± 16.1	−7
PCS (0–52)	Baseline	14.3 ± 10.7	
	T0	11.4 ± 8.6	−2.9
	T1	5.8 ± 6.5	−8.5
	T2	6.3 ± 7.9	−8

SPADI, Shoulder Pain and Disability Index; TSK-11, Tampa Scale for Kinesiophobia; CSI, Central Sensitization Inventory; PCS, Pain Catastrophizing Scale; MD, mean difference. #: significantly different between baseline and follow-up, *p* < 0.05.

**Table 3:** Self-reported shoulder pain and range-of-motion outcomes at baseline, two-week “washout” period (T0), posttreatment (T1), and follow-up (T2).

		Mean ± SD	MD
NPRS (0–10)	Baseline	2.6 ± 1.9	
	T0	2.9 ± 1.8 *	0.3
	T1	1.4 ± 1.1 †	−1.2
	T2	0.3 ± 0.4 #	−2.3
PER ROM (degrees)	Baseline	27.6 ± 19.6	
	T0	32.4 ± 25.9	4.8
	T1	30.9 ± 22.3	3.3
	T2	40.6 ± 24.4	13
AF ROM (degrees)	Baseline	106.6 ± 34.4	
	T0	105.8 ± 32.1 *	−0.8
	T1	120.1 ± 35.3 †	13.5
	T2	138.3 ± 33.1 #	31.7

NPRS, numeric pain rating scale; PER ROM, passive external rotation range of motion; ASF ROM, active shoulder flexion range of motion; MD, mean difference. *: significantly different after treatment compared to baseline; †: significantly different between post-treatment and follow-up, *p* < 0.05; #: significantly different between baseline and follow-up, *p* < 0.05.

**Table 4:** TPDT and laterality judgement at baseline, two-week “washout” period (T0), post-treatment (T1), and follow-up (T2).

			Mean ± SD	MD
TPD threshold		Baseline	42.5 ± 29.9	
		T0	35.8 ± 26.1	−6.7
		T1	28.1 ± 11.5	−14.4
		T2	27.5 ± 11.5	−15
Laterality judgement (right shoulder)	Accuracy (%)	Baseline	86 ± 11.03	
		T0	90 ± 16.6	4
		T1	95.9 ± 5.9	9.9
		T2	96.6 ± 5.01	10.6
	Speed (s)	Baseline	1.5 ± 0.3	
		T0	1.4 ± 0.3	−0.1
		T1	1.3 ± 0.2	−0.2
		T2	1.4 ± 0.2	−0.1
Laterality judgement (left shoulder)	Accuracy (%)	Baseline	93.9 ± 8.7	
		T0	94.6 ± 5.2	0.7
		T1	99.1 ± 2.5	5.2
		T2	93.3 ± 11.2	−0.6
	Speed (s)	Baseline	1.8 ± 0.4	
		T0	1.8 ± 0.7	0
		T1	1.6 ± 0.5	−0.2
		T2	1.4 ± 0.3	−0.4

TPDT, Two Point Discrimination Threshold; MD, mean difference.

## Data Availability

The datasets used and analysed during the current study are available from the corresponding author on reasonable request.
